# Care ethics and the transformation of care in an age of artificial intelligence

**DOI:** 10.1186/s12910-026-01474-8

**Published:** 2026-05-30

**Authors:** Rachel Wangari Kimani

**Affiliations:** 1 Department of Law, School of Social Sciences, The University of Manchester, Oxford Road, Manchester, M13 9PL UK; 2 Decker College of Nursing and Health Sciences, Binghamton University, Binghamton, NY USA

**Keywords:** Care ethics, Artificial intelligence, Healthcare robotics, Moral responsibility, Relational autonomy, Bioethics and technology

## Abstract

**Background:**

The integration of artificial intelligence (AI) and care robots into healthcare raises a central ethical question: what constitutes care, and how should it be delivered when machines perform caregiving tasks? Dominant AI ethics frameworks, including principlism, deontology, and consequentialism, focus on fairness, duties, and outcomes. While important, these approaches often view care primarily as technical compliance or efficiency, overlooking its relational and evaluative aspects.

**Methods:**

This analysis employs a normative, conceptual approach rooted in care and relational ethics, examining duty-based, outcome-oriented, and virtue-based care frameworks to highlight how they differ from care ethics as a structured moral practice. Tronto’s four stages of care (attentiveness, responsibility, competence, and responsiveness) serve as a framework for assessing moral labor in caregiving. Literature on social robots in healthcare is used illustratively. Conceptual analysis compares the interpersonal and moral dimensions of human caregiving with forms of AI interaction, noting changes when machines mediate or perform caregiving tasks.

**Results:**

The analysis shows that although AI systems can improve monitoring, coordination, and task performance, they do not assume moral responsibility or provide the relational and evaluative work that caregiving requires. Social and assistive robots reorganize moral labor by shifting attentiveness toward sensing, responsibility toward oversight, competence toward optimization, and responsiveness toward adaptive feedback. These changes create a functional resemblance to care without reproducing the moral engagement that characterizes genuine caregiving.

**Conclusion:**

Care ethics elucidates the moral practices of caregiving and how these practices are transformed through the integration of AI into healthcare relationships. Since caregiving involves vulnerability, interdependence, and judgment, it cannot rely solely on efficiency. A care-ethical perspective demonstrates that AI does not replace moral labor; rather, it reorganizes it in ways that reduce the conditions under which authentic caregiving can be conducted. The incorporation of care ethics into AI governance frameworks provides tools for assessing not just what these technologies do but what they cost the moral practice of caregiving.

## Introduction

As healthcare systems face mounting pressures from demographic changes, the growing prevalence of chronic diseases, and persistent workforce shortages, artificial intelligence (AI) is increasingly proposed as a solution [1]. These technologies are promoted as delivering efficiency, accessibility, and relief for overstretched caregivers, and surveys suggest that patients and professionals are broadly receptive [2, 3]. To date, most AI applications have been administrative or assistive; automating clinical documentation, streamlining billing, optimizing scheduling, and supporting supply chains [4]. Alongside these administrative functions, there are notable clinical tools, including the Da Vinci surgical system for minimally invasive procedures, decision-support algorithms that assist in radiology and pathology, and AI-enabled monitoring systems that flag early signs of patient deterioration [5]. While such systems raise questions of safety, accountability, and access, they largely remain technical supports [6]. They augment clinicians’ capacities but have yet to embody caregiving itself.

Philosophical perspectives on AI challenge this instrumental framing. Floridi (2013, 2018) situates AI within an emerging infosphere, a shared moral and informational environment in which humans and intelligent systems co-act as ethical participants [7, 8]. On this account, AI is more than a technical instrument; it represents a new form of intelligent agency that shapes moral decision-making and the negotiation of trust, responsibility, and dignity. In healthcare, these stakes are particularly high. Caregiving relationships are built on vulnerability, responsibility, and trust. Vulnerability here encompasses not only patient fragility but the broader human condition of dependency and exposure, which gives care its moral weight [9].

Social robots are increasingly deployed in healthcare for companionship, rehabilitation, mobility assistance, and telepresence [10]. Nieto Agraz et al. identify over 100 such systems in nursing, documenting tensions between autonomy and dependency, efficiency and presence, and professional roles and technological mediation [11]. These tensions raise a deeper question: what do we give up when structural pressures such as workforce shortages, cost constraints, and demographic demands make robotic care appear not just convenient but necessary? The answer reaches beyond efficiency. What is at stake is the moral character of caregiving itself — something Montemayor argues is beyond AI’s reach in principle. True empathy requires consciousness, embodiment, and mutual vulnerability [12]. Machines possess none of these.

AI systems may offer supportive benefits in resource-limited settings. But the ethical concern is not whether they work; it is what they quietly do to moral responsibility. By mimicking care, they shift the locus of moral labor without making that shift visible or accountable. This places the human caregiver in what Elish calls a “moral crumple zone”: absorbing accountability for algorithmic failures and caregiving deficits that the system cannot detect or repair [13]. The clinician bears responsibility for failures that originate upstream in design, deployment, and institutional decision-making.

This displacement is the core problem this paper sets out to analyze. Existing ethical and regulatory frameworks can articulate thresholds of safety and accountability [14, 15], but they operate primarily at the level of system design and outcomes. They are unable to track how moral responsibility shifts across the stages of recognizing, responding to, and maintaining needs. Tronto’s care ethics offers a more adequate framework precisely because it treats caregiving as a structured moral practice rather than merely a technical function. The question, then, is not whether AI can simulate care, but how its integration reconfigures the moral labor that caregiving requires.

Several scholars have examined this redistribution of moral labor from different angles. Van Wynsberghe’s research on care-centered design demonstrates how robotic systems can be developed in alignment with care values [16]. Coeckelbergh assesses artificial agents against normative care criteria, examining whether they can sustain relational interaction, embodied skills, and meaningful human contact practices [17]. Feminist and relational ethics scholars have situated care robots within broader debates about dependency, gendered labor, and structural responsibility [18, 19]. Vallor goes furthest, examining how technological systems shape the cultivation of moral character and practical wisdom in everyday caregiving contexts [20, 21],

What remains largely unexamined is how AI integration impacts caregiving as a structured moral practice. Beyond shaping values or outcomes, it redistributes the specific stages through which needs are identified, assumed, responded to, and maintained, namely [22] phases of attentiveness, responsibility, competence, and responsiveness. Elish and Hwang’s study of aviation autopilot litigation is instructive: even as control in complex systems is shared among humans and intelligent technologies, social and legal accountability continues to focus on the nearest human actor [23]. In healthcare, the moral crumple zone operates through the redistribution of moral responsibility across Tronto’s phases, not through system design or regulation. This is the gap the present analysis addresses.

This article argues that integrating AI transforms the moral dimension of caregiving by influencing how values are held, and virtues are cultivated, as well as how Tronto’s phases are implemented in practical caregiving contexts. For example, consider an elderly person with dementia whose agitation diminishes in the presence of a robot designed to detect distress and respond with calming sounds and movements. This robot performs as intended, and the patient becomes calm. This calmness is often seen as evidence of effective care, but it masks what has been quietly replaced: the relational work of a human caregiver, substituted by an algorithm programmed to handle agitation. The loss is not immediately visible in the outcome but lies in the moral labor that makes care a practice rather than just a function. Using Tronto’s framework, this analysis assesses how existing ethical theories address and sometimes overlook these transformations, arguing that care ethics is particularly suited to understanding their moral significance.

The argument develops in six sections. The first situates AI-mediated caregiving within its ethical and regulatory context. The second clarifies what care is and what gives it moral significance. The third examines how deontological, consequentialist, and virtue ethics approach technologically mediated caregiving; the fourth identifies where each falls short as AI assumes caregiving roles. The fifth advances Tronto’s care ethics as a more adequate framework. The sixth applies this to social robots, showing both the possibilities and limits of technological caregiving.

### Ethical and regulatory context of AI-mediated care

To clarify the stakes of this analysis, it is necessary to examine how AI in healthcare is framed within contemporary ethical and regulatory discourse. Governance approaches typically evaluate AI with respect to risk management, safety, accountability, and professional oversight, thereby structuring these systems as technical instruments that require mitigation and supervision [24, 25]. Yet even where patients and professionals express openness to AI-mediated care, this acceptance does not resolve whether simulated empathy or algorithmic responsiveness can sustain the moral and relational core of caregiving [12, 26].

The European Union’s AI Act exemplifies this approach. Drawing on Floridi’s concept of a Good AI Society, the Act embeds ethical principles, including beneficence, non-maleficence, autonomy, justice, and explicability, into the design and governance of AI systems. Explicability, Floridi’s distinctive addition to the traditional bioethical canon, combines intelligibility and accountability to answering both “how does this AI system work?” and “who is responsible for the way it works?” [27]. Floridi argues that responsibility cannot be assigned unless the system is understood. Together, they form the ethical infrastructure that the EU calls “trustworthy AI.” [7]. Article 1(1) of the EU affirms the Act’s dual aim: to promote AI development while protecting individuals and society from harm.

In contrast, U.S. responses remain fragmented. Illinois’s Wellness and Oversight for Psychological Resources Act prohibits AI from delivering psychotherapy unless licensed professionals are in charge, an effort prompted by harmful chatbot interactions [28]. Similarly, California’s Assembly Bill 489 forbids AI systems from implying affiliation with licensed healthcare professionals without genuine oversight [29]. Both laws target representational deception, recognizing that in healthcare, moral harm can arise not only from erroneous decisions but from misleading relationships of trust. That such concerns have entered legislation signals a growing policy recognition that the integrity of the caregiving relationship matters. This is precisely the concern care ethics foregrounds. A system that simulates trustworthiness without genuine commitment does not merely deceive; it undermines the conditions under which care can be ethically sustained. Together, these state-level initiatives reveal a distinctly American pattern of piecemeal governance: protective of professional boundaries yet lacking a unified moral framework.

European regulation embeds macro-ethical principles; U.S. policy safeguards liability. Neither addresses the interpersonal dimensions of care that care ethics identifies as foundational. These divergent policy and regulatory orientations reveal a structural limitation. Governance can articulate thresholds of safety and responsibility, but it cannot determine what constitutes good care. Even where AI systems are explainable, supervised, and legally compliant, the question remains whether they sustain the relational and moral practices through which care is enacted. To address this question, it is necessary to clarify what care is and how its ethical quality can be evaluated.

### What is care?

Care in healthcare encompasses physical interventions, hygiene practices, documentation procedures, and the coordination of clinical routines. Care is more than task execution. It is an interpersonal and institutional practice focused on maintaining others’ well-being [30]. Krause and Boldt characterize it as *“a set of relational actions that occur within an institutional context and aim to maintain*,* improve*,* or restore well-being*,” emphasizing that care is intentional and goal-directed rather than routine or procedural [31]. Mol similarly holds that care is inseparable from context and from the shifting needs of patients [32].

Tronto and Fisher extend the concept further, describing care as *“everything we do to maintain*,* continue*,* and repair our world so that we can live in it as well as possible.”* This formulation situates care not only in clinical or domestic settings but as a species-wide activity encompassing relationships, institutions, and environments [33]. For Held, care is both a practice and a value, that establishes a moral foundation comparable to justice or autonomy [34]. Across these views, care emerges as ethical, relational, and political.

Care ethics, as articulated by Tronto, is part of a broader group of approaches, including feminist and relational ethics, which all emphasize dependency and interpersonal relationships as the moral foundation. These approaches differ in emphasis: feminist ethics highlights gendered labor and political systems, whereas care ethics centers on the moral act of caregiving itself. When the term “relational” is used here, it refers to the bonds of dependency, vulnerability, and moral responsiveness between caregiver and patient, embedded within the organizational and structural contexts that shape how care is practiced.

Situating care within this relational and political framework is significant because care has traditionally been marginalized as a feminized, private labor rather than acknowledged as a political priority. Tronto shows how care is frequently devalued, outsourced, and rendered invisible. She distinguishes between “small-p politics,” the everyday, interpersonal practices of caregiving, and “Big-P Politics,” which encompasses the structural arrangements, economic systems, and policies that shape how care is distributed and valued [35]. Care is not confined to the bedside or home. It involves political institutions and the broader economy. Tronto’s contribution is especially significant here because she offers not only a broad account of care but a way to assess its ethical quality.

Tronto’s care ethics framework thus serves both descriptive and evaluative purposes, reflecting the two senses in which “care” is used throughout this paper: as an activity (what caregivers do) and as a moral standard (how well they do it). It is descriptive because it outlines the stages in care (attentiveness, responsibility, competence, and responsiveness) and provides vocabulary for understanding how care is performed in everyday situations [36]. It is evaluative because each phase acts as a benchmark for determining whether care meets ethical standards. A caregiver might recognize a patient’s need and feel responsible for responding, yet still fall short if the care is poorly delivered or fails to attend to the patient’s experience. This dual role enables assessment of both caregiving actions and the institutional frameworks, including AI, that shape and distribute care.

Care is dynamic, continually reshaped by social, political, and technological forces. AI integration raises questions beyond safety or efficiency; it changes the conditions under which care is practiced, valued, and distributed [37]. Assessing these technologies requires attention to their impact on the moral dimensions of care, not just their technical performance. The following section examines the principal ethical traditions of deontology, consequentialism, and virtue ethics to situate care ethics within a broader normative context.

### Ethical frameworks on care: duty, outcome, and virtue

If care is understood as a relational, moral, and political practice, the next step is to ask how other ethical traditions account for it. In bioethics, deontological, consequentialist, and virtue approaches remain dominant, shaping professional codes, institutional policy, and the ethical evaluation of emerging technologies. Each provides important resources for assessing AI in healthcare: duties foreground fairness and obligation, outcomes emphasize measurable benefit and harm, and virtue ethics attends to character and practical wisdom.

While these frameworks illuminate important dimensions of ethical analysis, they tend to treat relationality as derivative rather than foundational. They evaluate actions, outcomes, or agents; they do not typically take the structure of relational practice itself as the primary locus of moral concern. Examining each in turn clarifies both their strengths and their limits when applied to AI-mediated care.

### Care as duty (deontological ethics)

Deontological ethics, particularly in its Kantian form, views care as a duty to universal principles, prioritizing impartiality and consistency over contextual or emotional engagement [38, 39]. This has shaped professional ethics in medicine, law, and nursing, and finds its most influential bioethical expression in Beauchamp and Childress’s principlist framework, grounded in autonomy, beneficence, non-maleficence, and justice [40].

AI systems seem well-suited to this approach: they apply fixed rules consistently and without emotional bias, aligning with the deontological ideal of impartial, duty-driven care [41, 42]. However, this also reveals a structural limitation. Deontological models assume that moral duties can be fully defined in advance and reliably carried out by accountable agents. This assumption breaks down in situations that demand moral flexibility and contextual judgment, where rigid adherence to universal principles is ethically insufficient [43, 44]. More fundamentally, unlike human caregivers, who can be held responsible for moral failures, AI systems cannot be held accountable for the harm caused by their errors, undermining the trust essential to caregiving.

### Care as virtue and practical wisdom (virtue ethics)

Virtue ethics conceptualizes caregiving as a moral endeavor that requires cultivated character and practical wisdom (phronesis) [45, 46]. This is understood as an embodied sensitivity to others’ needs, vulnerabilities, and particularities that cannot be reduced to rules or outcomes [47]. Unlike duty-based or outcome-based accounts, ethical action here requires judgment formed through experience, reflection, and interpersonal practice. AI systems lack phronesis and the capacity for character development; they cannot cultivate virtue or exercise reflective judgment.

For this reason, scholars such as Shannon Vallor argue that virtue ethics remains relevant to AI not as a model of machine agency but as a guide for human designers and institutions [48]. Her account of “technomoral virtues” emphasizes that the ethical quality of AI depends on the character and practical wisdom of those who design, deploy, and govern it. Since virtue ethics concentrates moral evaluation on the development and excellence of agents within practices, its principal analytical focus remains on the character and judgment of caregivers. AI does not become virtuous; rather, it reflects the moral formation of its creators.

However, when AI systems are integrated into care, the ethical issue extends beyond character formation to the reorganization of the conditions under which judgment is exercised. AI does more than assist virtuous agents; it reshapes the environment in which practical wisdom operates [49]. For example, decision-support tools might limit clinical judgment, algorithmic prompts could shift what is deemed important, and automated triage might prioritize whose needs are addressed first. In such cases, optimization displaces the negotiation of need between caregiver and patient. Thus, the challenge is not simply a lack of machine phronesis but a fundamental reorganization of caregiving itself.

### Care as an outcome (consequentialist/utilitarian ethics)

Consequentialist ethics evaluates care by its outcomes, seeking to maximize well-being, minimize harm, and allocate resources efficiently [50]. From this viewpoint, AI is particularly appealing: diagnostic tools lower errors, monitoring devices help prevent crises, and elder-care robots expand coverage in areas with staff shortages [51]. In this framework, the moral worth of caregiving depends on its results rather than the caregiver’s character or the quality of the caregiving relationship [52]. Moreover, unlike human caregivers, AI systems do not tire, experience mood swings, or need breaks, and they can consistently follow guidelines at scale.

However, this outcome-based lens risks reducing care to measurable benefits while overlooking its moral dimensions. Patients may receive technically successful treatment yet still feel unseen or dehumanized, what Krause and Bolt describe as “thin care” [31]. AI further exposes structural tensions in consequentialism: predictive algorithms may optimize for aggregate outcomes yet perpetuate systemic inequities when trained on biased data. For instance, studies of clinical risk scores have shown that algorithms used to allocate resources often disadvantage marginalized groups by encoding historical disparities [53]. When caregiving is evaluated primarily by aggregate outcomes, the moral significance of attentiveness, vulnerability, and human presence becomes secondary. Care is reframed as optimization rather than as a practice embedded in dependency and moral commitment.

### Transformation of care: what happens when AI becomes the caregiver?

Each of the dominant ethical traditions illuminates an important dimension of caregiving. Deontological frameworks safeguard fairness and consistency; consequentialist reasoning tracks measurable benefits and harms; virtue ethics emphasizes the cultivation of moral character and practical wisdom. When applied to AI, these approaches help explain the appeal of machine-mediated care: algorithms can reliably follow rules, optimize outcomes at scale, and support human professionals in exercising structured judgment.

Yet each also reveals a limitation. Duty-based systems struggle when moral flexibility and contextual sensitivity are required. Outcome-based reasoning risks reducing care to metrics, overlooking lived experience and human presence. Virtue ethics locates moral authority in cultivated human judgment, leaving unresolved the question of how non-human systems participate in or reshape that formation. In each case, caregiving is partially abstracted from the interpersonal practices through which needs are recognized, negotiated, and responded to.

Care ethics shares common ground with these traditions by incorporating ideas of responsibility, harm, justice, and practical wisdom. Its unique contribution is to view the structure of caregiving itself as the main focus of moral concern, thus analyzing not only whether duties are performed or virtues are developed, but also how attentiveness, responsibility, competence, and responsiveness are enacted in caregiving relationships. Unlike other approaches that assess care externally, care ethics investigates it from within, making it particularly well-suited to tracking how AI integration redistributes moral labor across the phases of caregiving.

### Care ethics and AI caregiving

Joan Tronto’s care ethics is especially pertinent in the era of artificial intelligence, where machines are more frequently taking on caregiving roles. Although AI is often presented as a remedy for staff shortages and cost concerns [54], Tronto’s framework questions whether such technologies align with the ethical commitments of care or deepen the structural devaluation of caregiving labor. As noted earlier, Tronto’s distinction between small-p and Big-P politics operates at two levels. At the interpersonal level, AI can appear to enhance care through health monitoring, task assistance, and companionship. At the systemic level, its development is driven by political and economic priorities that have long marginalized care work.

Tronto identifies four interrelated phases: of care: attentiveness, responsibility, competence, and responsiveness [30]. Applied to AI, these phases illuminate how the integration of algorithmic systems reshapes the moral structure of caregiving. AI can augment attentiveness through sensing technologies and support competence through standardization and reliability. At the same time, responsibility becomes distributed across designers, clinicians, institutions, and regulatory actors, complicating traditional lines of accountability. Responsiveness, meanwhile, is mediated by programmed feedback rather than by reciprocal vulnerability; machines can register input but do not experience moral concern [55]. These shifts move the discourse beyond whether machines “care” to how care itself is reconfigured through algorithmic mediation.

Empirical research supports this distinction. Studies show that patients may initially rate AI-generated responses as highly empathetic; however, once informed that the responses were machine-generated, trust and emotional satisfaction decline [56]. Similarly, Rubin et al. argue that because AI’s expressions of empathy do not involve emotional labor, they fail to convey genuine care, which patients often perceive as a sign of their unique worth [57]. Kurian also identifies an “empathy gap” in conversational AI, noting that simulated responsiveness can leave vulnerable users, especially children, feeling invalidated or unsupported [58].

What appears to shift, then, is not functional adequacy but the relational meaning attached to emotional effort. When vulnerability is absent on the caregiver’s side, care does not disappear; rather, it assumes a different moral form. Trust, recognition, and alliance may still be produced, but through technical mediation rather than reciprocal exposure. The question is not whether care remains possible, but how its structure changes when one party cannot be affected in return.

Care ethics, as articulated by Tronto and developed across feminist and relational scholarship, provides criteria for evaluating caregiving practices, grounding moral judgment in the recognition of vulnerability, the assumption of responsibility, the exercise of competent response, and attentiveness to how care is received [18, 34, 59]. These dimensions constitute the moral architecture of care. The benchmark, therefore, is neither biological humanity nor current institutional habit, but the conditions under which caregiving remains responsive to vulnerability.

Caregiving’s moral architecture is increasingly shaped through technological mediation, a development widely analyzed in contemporary scholarship. Van Wynsberghe’s care-centered value-sensitive design applies Tronto’s framework to robotic care, showing that such systems “reconfigure” rather than reproduce moral relations [16]. Coeckelbergh further shows that moral life already depends on perceived emotional presence rather than direct access to inner states [60]. If robots convincingly simulate these appearances, they may be treated as moral participants regardless of their internal constitution. This does not establish robots as moral agents. It reveals how easily simulation displaces moral perception. Floridi calls this semantic pareidolia: past a threshold of similarity, we stop distinguishing and start attributing genuine care to the artifact [61].

In caregiving contexts, this mediation assumes institutional and practical form. AI systems do not merely supplement existing roles; they alter how vulnerability is identified, how professional skill is exercised, and how accountability is distributed across clinical and technological infrastructures [62]. Sensing technologies have the potential to augment attentiveness through the detection of patterns imperceptible to human perception. Algorithmic systems may increase consistency by standardizing tasks and minimizing variability. Consequently, moral accountability is no longer confined to the caregiver–patient interaction but extends across networks of design, implementation, and oversight.

This configuration may be characterized as engineered care: caregiving practices systematically organized through data processing, predictive modeling, and technological mediation. This term does not suggest that such engineered care is a substandard imitation of human care. Rather, it marks a distinct arrangement in which interpersonal engagement is partially structured by technical systems, rather than sustained solely by reciprocal human presence. Care ethics offers a framework for evaluating this arrangement without romanticizing traditional practice or uncritically embracing technological efficiency.

The ethical focus, then, is on how care’s moral form changes when one participant cannot be affected in return. Under technological mediation, attentiveness becomes data-driven, competence standardized, responsibility distributed, and responsiveness simulated. Social robots make this transformation especially visible. As embodied systems that emulate responsiveness while remaining insulated from reciprocal vulnerability, they provide a focused site for examining how caregiving’s moral architecture is reshaped.

### Care ethics applied: social robots as a testing ground

**Fig. 1:**
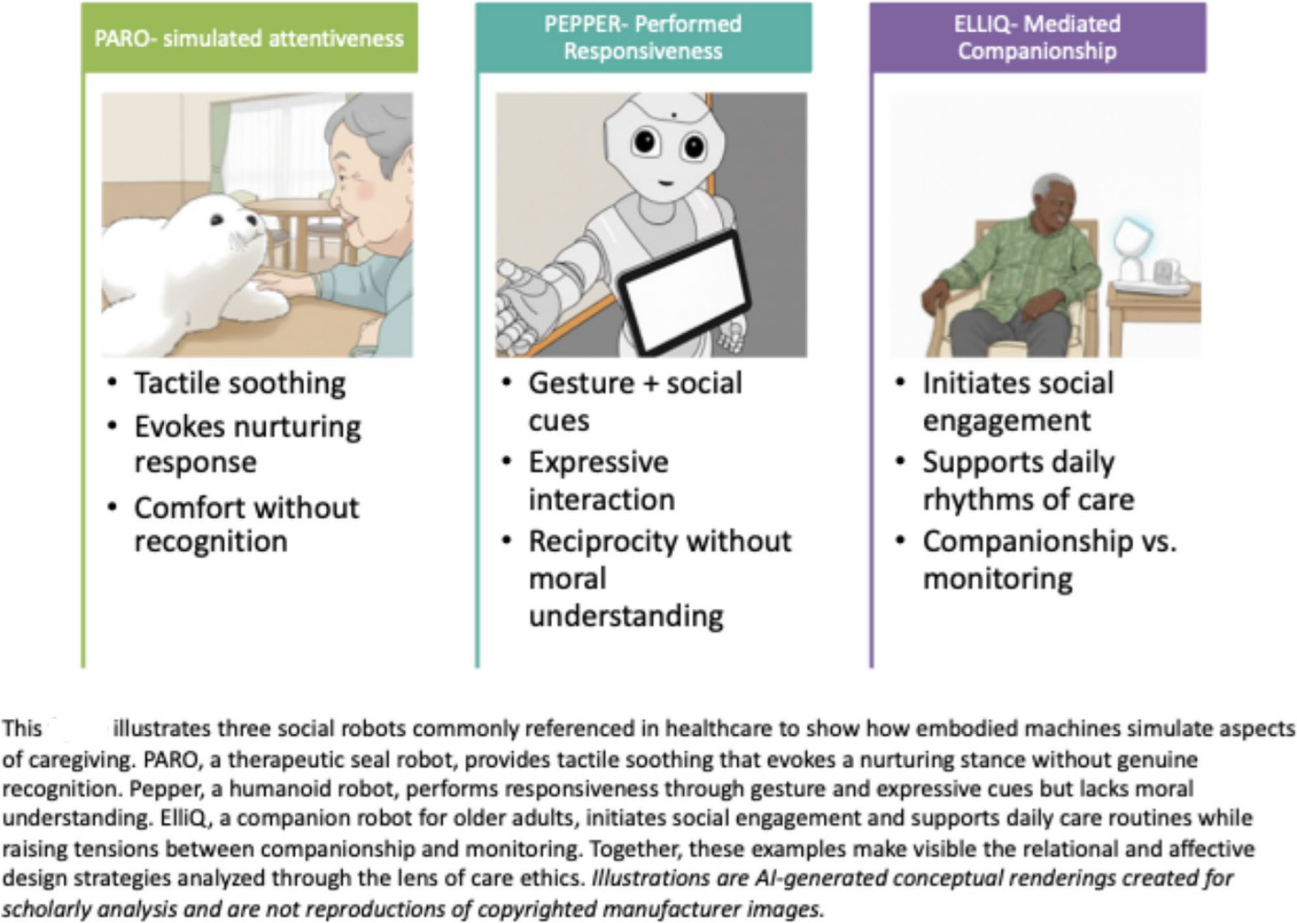
Social robots in care settings

Social robots, as defined in Soraa et al., are machines characterized by their ability to “portray social abilities to assist or support humans socially” [63]. Their increasing deployment spans sectors such as healthcare, education, and rehabilitation. As Ragno et al. expound, these robots are not merely service tools but “artificial systems capable of playing an active and positive social role within a society in which human and non-human agents are present” [64]. This framing situates social robots beyond technical artifacts but as participants within relational environments where moral expectations arise. Figure 1 illustrates three widely used social robots whose design strategies illuminate these ethical tensions.

The ethical implications of social robots and embedded artificial intelligence must be carefully considered, even as their rapid advances bring tangible benefits to clinical practice. In older-adult care, social robots are used to enhance independence, support daily routines, reduce caregiver burden, and facilitate remote communication with family members [63, 65, 66]. Robotic pets, a common subtype, resemble companion animals and are increasingly deployed as therapeutic tools in pediatric and geriatric contexts [67, 68]. In children’s hospitals, robotic companions can distract from pain and fear; in long-term care, robotic pets provide comfort and help sustain routines when human attention is limited.

These deployments frequently occur under conditions of workforce shortage, demographic aging, and institutional strain. In many regions, persistent gaps in nursing and caregiving staff have led to social robots being framed as pragmatic responses to structural scarcity or as tools intended to mitigate caregiver burden and compensate for limited staff availability [69–71]. For example, the large-scale deployment of Hyodol companion dolls in South Korea has been explicitly justified as a response to severe shortages in elder care, particularly among socially isolated older adults [72]. Social robots, therefore, emerge within systems already shaped by political and economic pressures that constrain sustained human presence.

Care ethics does not ignore these constraints. As Tronto’s small-p and Big-P politics remind us, caregiving has long been shaped by political and economic forces that undervalue care work [35]. Similarly, analyses of care robotics within feminist and relational ethics have warned that technological “solutions” to care deficits may conceal the structural devaluation of care labor and reframe expectations regarding human presence and responsibility [18, 19]. Robert and Linda Sparrow further argue that introducing machines into intimate care contexts may subtly redefine what counts as adequate care and reshape the moral landscape of dependency [73].

It is precisely because these technologies effectively evoke relational responses that they give rise to complex moral questions. Their apparent attentiveness and empathy prompt us to scrutinize the true nature of “care” practiced when a machine assumes the role of a companion or caregiver. Consequently, they illuminate the redistribution of moral labor among human and technical actors. This redistribution becomes more evident when each phase of care, attentiveness, responsibility, competence, and responsiveness is examined in turn.

### Attentiveness

Tronto defines attentiveness as the capacity to notice another’s need and to understand that need as generating a moral claim on the caregiver [74]. This involves both perceptual awareness and an ethical orientation toward the other. In care ethics, attentiveness is therefore not a passive act of sensing but an active openness to being addressed by another’s vulnerability. By contrast, AI systems treat attention as an informational task: detecting cues, ranking inputs, and generating optimized outputs. In human–robot interaction, this difference becomes evident when robots simulate attentiveness through pre-programmed gaze patterns, movements, or scripted speech. These responses register signals but do not constitute the moral attentiveness Tronto describes.

Social robots such as Paro and Pepper illustrate this tension. They detect emotion through touch, tone, and expression, enabling adaptive responses that comfort or engage users [75]. Research shows that such robots can calm agitation and promote engagement in dementia patients, yet their behavior is reactive rather than interpersonal [65]. They respond based on preset stimuli rather than recognizing the individual in front of them. The same comforting response is given regardless of the person’s identity, reasons, or needs. The comfort they offer arises from mimicry, not moral understanding. Similarly, Pepper, a humanoid robot equipped with cameras and microphones, interprets facial expressions and tone of voice to adjust its replies [68]. However, its output reflects pattern-matching rather than any evaluative grasp of another’s situation.

The opacity inherent in these systems compounds this deficit. When AI responses are neither comprehensible nor interpretable, caregivers and institutions risk conflating algorithmic reaction with genuine attentiveness. This is not a hypothetical concern; AI monitoring systems currently enable nurses to monitor patients via sensors and wearable devices remotely, thereby reducing the need for direct bedside care [76, 77]. Presented as efficiency enhancements, these setups subtly reshape the concept of attentiveness in real practice. As the system shifts from being a mere tool to acting as a surrogate judge of need, caregivers slowly lose their moral and perceptual ability to identify needs beyond its preset parameters, much like a clinician who depends only on test results stops developing the clinical judgment to recognize what tests miss [78]. What initially starts as technological aid gradually becomes the norm for defining need itself.

When robots assume the task of sensing, the moral labor of attentiveness is redistributed among human and non-human actors. The caregiver’s role shifts from perceiving need to interpreting data, while designers and institutions quietly determine what qualifies as “need” and how it should be measured. In this configuration, attentiveness becomes an operational feature of the system rather than a moral stance.

### Responsibility

Attentiveness begins the moral act of care by recognizing need, with responsibility guiding that recognition. Tronto states responsibility is where perception turns into commitment—making another’s need a moral concern (Tronto, 1993). In human caregiving, responsibility is personal and professional, based on trust, responsiveness, and moral agency, and is interpersonal rather than procedural: one takes responsibility for a person, not just a task. This relational orientation links responsibility to the ongoing moral relationship, not merely to the execution of discrete duties.

In AI caregiving, responsibility is distributed among designers, clinicians, institutions, and algorithms, creating what Elish calls a “moral crumple zone,” in which accountability is assigned to the nearest human when systems fail [13]. Social robots illustrate this redistribution: their actions depend on programming and context, but their presence generates moral expectations. Who is accountable when a robot comforts someone: the designer, the operator, or the institution? The answer is all of them, and therein lies the problem. When responsibility is distributed across an entire network of actors, it becomes nobody’s specific moral concern. The crumple zone operates precisely here: not through negligence but through a system that functions as designed while a particular person’s need goes unrecognized and unaddressed.

Van Wynsberghe extends this analysis by emphasizing that responsibility also concerns the caregiver’s stance or commitment to ensuring that the cared-for is directed toward the appropriate form of care and that their needs are accurately assessed [16]. As technologies mediate these assessments, elements of this evaluative responsibility become delegated to artifacts and infrastructures. Verbeek’s notion of technological mediation becomes central here: decision-making in healthcare is now a hybrid affair between clinician, patient, and machine [37]. Algorithms triage alerts, recommend treatments, or determine what counts as relevant information, subtly shaping how human caregivers perceive and act. Schiff describes this moral handover of AI documentation systems that summarize clinical encounters [78]. Designed to relieve administrative burden, these tools often strip narrative and emotional nuance from patient–provider dialogue, producing efficient but impersonal records. The result is a shift in who identifies need, what information is preserved, and which aspects of the encounter become morally salient.

Care ethics views responsibility as an ongoing moral relationship rooted in dependency and trust [34], rather than a fixed duty assigned to individuals or organizations. When design choices and institutional structures determine what constitutes a need that warrants response, this ongoing connection is disrupted. A system configured to recognize certain signals cannot identify needs outside its scope. Consequently, individuals whose distress remains unnoticed become morally invisible — not because anyone intentionally neglects them, but because the system was never built to perceive them. This issue is more than philosophical: pulse oximeters, validated mainly on lighter skin, have been shown to systematically overestimate oxygen levels in patients with darker skin, leaving critical deterioration undetected until it becomes life-threatening [79].

### Competence

Tronto’s third phase of care, competence, requires that care not only be well-intentioned but also effectively meet needs, integrating moral commitment with the technical skill and practical wisdom required to respond appropriately. In human caregiving, competence involves judgment, contextual awareness, and adaptability to the patient’s lived experience. It refers to the capacity to identify what good care requires in a particular situation and to carry it out in a way that supports the other’s well-being.

In AI-mediated caregiving, competence is typically evaluated through performance metrics such as speed, accuracy, and reliability. Robots often excel at these measures, yet their competence is operational rather than moral. AI systems function within predefined parameters and cannot apprehend the ambiguity, narrative complexity, or evaluative meaning of human suffering, nor do they possess the motivation to alleviate it [80]. They can complete tasks but cannot exercise discernment about what constitutes good care in a particular moment for a particular person. As González and Iffland note, care, by its very nature, is particular; it does not concern abstract humans but focuses on the specific historical and geographical details of a particular individual [81].

For example, a medication administration system can dispense the appropriate drug at the correct dosage; however, it lacks the capacity to assess whether the patient is sufficiently well to receive it at that particular time. Determining whether their condition has altered, if they appear more confused than the previous day, or if something feels off that hasn’t yet been reflected in the data. Such judgment entails synthesizing protocol knowledge with direct observation, interpersonal attentiveness, and a moral obligation to the patient’s well-being.

The development of systems capable of integrating multiple physiological data streams suggests that AI may eventually bridge this gap. However, even a system capable of measuring pupils, identifying distress signals, and administering appropriate medication remains fundamentally different from clinical judgment. It can only recognize patterns within its training limits. It cannot identify what it has never encountered before. More fundamentally, as technical capabilities advance, the moral structure of competence is still reorganized. When the system errs, who bears the moral responsibility? A more advanced algorithm does not resolve this moral dilemma. It simply makes the problem harder to see.

Care competence, therefore, encompasses moral interpretation and the ability to promote human flourishing, aspects that cannot be automated, even as technical tasks are assigned to machines. When AI assumes the execution of care tasks, the question is not whether it performs them accurately, but whether the conditions for genuinely competent care, such as clinical judgment, contextual awareness, and interpersonal attentiveness, are preserved or gradually displaced.

### Responsiveness

Responsiveness in care emphasizes how the recipient perceives it. Tronto describes it as being attentive to the other’s responses, turning caregiving into a mutual dialogue of adaptation. AI caregiving replaces this moral exchange with iterative technical feedback loops. Robots such as Paro or Pepper “learn” user preferences through reinforcement algorithms and sentiment analysis, but this constitutes behavioral adaptation rather than evaluative understanding. Yuan and Coghlan describe such interaction as computational mimicry of empathy: systems detect affective cues and generate appropriate outputs, yet lack the moral reflection that genuine responsiveness entails [82]. Rubin similarly distinguishes between recognizing emotion and sharing it; empathy requires entering another’s emotional world, not merely mapping it [57].

What distinguishes genuine responsiveness from its simulation is vulnerability. A human caregiver can be influenced by what they experience from the patient, feeling moved, troubled, or personally changed by what they observe. That being-affected is not incidental to care; it is what transforms feedback into moral response. A robot can register a patient’s distress and generate a soothing reply. It cannot be affected in a way that changes its moral orientation toward that person. It processes without being present.

This distinction is especially clear with children. A child interacting with social robots that respond cheerfully and positively may learn to see these responses as real affection. Robots are always patient and available, unlike humans, who involve friction, misunderstandings, and vulnerability. When simulated responses replace genuine care, children learn a distorted view of care. Genuine relationships involve mutual vulnerability and can feel like failures if replaced by robotic interactions. Moral responsiveness is shaped by practice, and as Turkle shows, digital engagement alters expectations of human contact [83]. Children practicing responsiveness mainly with machines may struggle with real human relationships. Without friction, we lose the muscle for genuine connection [58].

AI integration ultimately produces a bidirectional reorganization of caregiving. As patients adjust to receiving simulated responsiveness, caregivers adapt to providing it, thus progressively narrowing what counts as care on both sides of the relationship. Attentiveness evolves into sensing, responsibility transforms into oversight, competence shifts into optimization, and responsiveness develops into adaptation. What is lost at each stage is not merely a human attribute but the moral framework that underpins caregiving and sustains the conditions necessary for human flourishing.

## Conclusion

AI is increasingly promoted as a solution to the caregiving crises produced by aging populations, workforce shortages, and the growing burden of chronic disease. These technologies promise efficiency, consistency, and expanded access, and they deliver on many of these promises. But efficiency is not care. This paper has argued that AI integration does not merely augment caregiving; it reorganizes the moral structure of caregiving in ways that are largely invisible, gradual, systemic, and easy to mistake for progress. The question is not whether these technologies work, but whether we have frameworks adequate to evaluate what they cost the moral practice of caregiving.

By tracing Tronto’s phases of attentiveness, responsibility, competence, and responsiveness through AI-mediated caregiving, this analysis has shown that each phase undergoes a characteristic transformation. Attentiveness becomes sensing as the moral act of recognizing a particular person’s need is replaced by data detection. Responsibility becomes distributed across networks of designers, institutions, and algorithms, creating conditions in which accountability belongs to everyone and therefore to no one. Competence is assessed based on operational metrics rather than moral judgment, thereby gradually displacing clinical discernment. And responsiveness becomes adaptation; simulated rather than genuine, processing rather than presence. AI integration ultimately produces a bidirectional reorganization: as patients adapt to receiving less, caregivers adapt to providing it, and the moral architecture of care contracts on both sides.

This analysis does not argue against technological innovation in healthcare. It argues for a more adequate framework to evaluate it. Principlist and regulatory approaches can establish standards for safety, accountability, and fairness, but they overlook what care ethics highlights: the gradual shift in moral responsibilities as machines take on caregiving roles. Before surrendering attentiveness to sensors, responsibility to algorithms, competence to optimization, and responsiveness to adaptive feedback, healthcare systems need frameworks that ask not only whether AI works, but what it costs the moral practice of caregiving. Tronto’s account of care as a structured moral practice offers precisely this: a way to evaluate not just what AI does, but what it undoes.

## Data Availability

No datasets were generated or analysed during the current study.

## References

[1] Maleki Varnosfaderani S, Forouzanfar M. The role of AI in hospitals and clinics: transforming healthcare in the 21st century. Bioengineering. 2024;11(4):337. 38671759 10.3390/bioengineering11040337 PMC11047988

[2] van Winkel SL, et al. AI as an independent second reader in detection of clinically relevant breast cancers within a population-based screening programme in the Netherlands: a retrospective cohort study. Lancet Digit Health. 2025;7(8). 10.1016/j.landig.2025.100882. 10.1016/j.landig.2025.100882 40816977

[3] Andtfolk M, et al. Attitudes toward the use of humanoid robots in healthcare—a cross-sectional study. AI Soc. 2022;37(4):1739–48.

[4] Reddy S. Generative AI in healthcare: an implementation science informed translational path on application, integration and governance. Implement Sci. 2024;19(1):27. 38491544 10.1186/s13012-024-01357-9 PMC10941464

[5] Bedaf S, Gelderblom GJ, De Witte L. Overview and categorization of robots supporting independent living of elderly people: What activities do they support and how far have they developed. Assist Technol. 2015;27(2):88–100. 26132353 10.1080/10400435.2014.978916

[6] Khan MM, et al. Towards secure and trusted AI in healthcare: a systematic review of emerging innovations and ethical challenges. Int J Med Informatics. 2025;195:105780. 10.1016/j.ijmedinf.2024.105780 39753062

[7] Floridi L, et al. AI4People—An ethical framework for a good AI society: Opportunities, risks, principles, and recommendations. Mind Mach. 2018;28(4):689–707. 10.1007/s11023-018-9482-5 PMC6404626 30930541

[8] Floridi L. The ethics of information. UK).ISBN: Oxford University Press; 2013. p. 0199641323.

[9] Boldt J. The concept of vulnerability in medical ethics and philosophy. Ethics Humanit Med. 2019;14(1):6. Philosophy. 10.1186/s13010-019-0075-6 PMC6458617 30975177

[10] Sørensen L et al. Humanoid Robots, A future health care service? In international conference on social robotics. 2024. Springer.

[11] Nieto Agraz C, et al. A survey of robotic systems for nursing care. Front Rob AI. 2022;9:832248. 10.3389/frobt.2022.832248 PMC9021873 35462781

[12] Montemayor C, Halpern J, Fairweather A. principle obstacles for empathic AI: why we can’t replace human empathy in healthcare. AI Soc. 2022;37(4):1353–9. 10.1007/s00146-021-01230-z. 34054228 10.1007/s00146-021-01230-z PMC8149918

[13] Elish MC. Moral crumple zones: Cautionary tales in human-robot interaction (pre-print). Engaging Science, Technology, and Society (pre-print); 2019.

[14] Montemayor C, Halpern J, Fairweather A. principle obstacles for empathic AI: why we can’t replace human empathy in healthcare. Volume 37. AI & society; 2022. pp. 1353–9. 4. 10.1007/s00146-021-01230-z PMC8149918 34054228

[15] Tavory T. Regulating AI in mental health: ethics of care perspective. JMIR Mental Health. 2024;11(1):e58493. 39298759 10.2196/58493 PMC11450345

[16] Van Wynsberghe A. Designing robots for care: Care centered value-sensitive design, in Machine ethics and robot ethics. Routledge; 2020. pp. 185–211. 10.1007/s11948-011-9343-6 PMC3662860 22212357

[17] Coeckelbergh M. Artificial agents, good care, and modernity. Theor Med Bioeth. 2015;36(4):265–77. 26002636 10.1007/s11017-015-9331-y

[18] Parks JA. Lifting the burden of Women’s care work: should robots replace the human touch? Hypatia. 2010;25(1):100–20.

[19] Gary ME. Care robots, crises of capitalism, and the limits of human caring. IJFAB: Int J Feminist Approaches Bioeth. 2021;14(1):19–48.

[20] Vallor S. Twenty-first-century virtue. Science, technology, and virtues: contemporary perspectives. 2021;77. 10.1093/oso/9780190081713.003.0005.

[21] Vallor S. The AI mirror: How to reclaim our humanity in an age of machine thinking. Oxford University Press.ISBN; 2024. p. 0197759084.

[22] Tronto JC. Moral Boundaries: A Political Argument for an Ethic of Care. United Kingdom: Routledge; 1993.

[23] Elish MC, Hwang T. Praise the machine! Punish the human! The contradictory history of accountability in automated aviation. Punish the human; 2015.

[24] Eldakak A, et al. Civil liability for the actions of autonomous AI in healthcare: an invitation to further contemplation. Humanit Social Sci Commun. 2024;11(1):1–8.

[25] Cheng L, Gong X. Appraising Regulatory Framework Towards Artificial General Intelligence (AGI) Under Digital Humanism. Int J Digit Law Gov. 2024;1(2):269–312. 10.1515/ijdlg-2024-0015.

[26] Johansson-Pajala R-M, Gustafsson C. Significant challenges when introducing care robots in Swedish elder care. Disabil Rehabil Assist Technol. 2022;17(2):166–176. 10.1080/17483107.2020.1773549 32538206

[27] Floridi L, Cowls J. A Unified Framework of Five Principles for AI in Society. Harv Data Sci Rev. 2019. 10.1162/99608f92.8cd550d1.

[28] Assembly IG. Public Act 104 – 0054: Wellness and Oversight for Psychological Resources Act, in Illinois Public Acts, 104th General Assembly. State of Illinois; 2025.

[29] California L. Assembly Bill 489: Health care professions: deceptive terms or letters: artificial intelligence, in Chap. 615, California Statutes of 2025. 2025, California State Legislature.

[30] Edwards SD. Three versions of an ethics of care. Nurs Philos. 2009;10(4):231–40. 19743967 10.1111/j.1466-769X.2009.00415.x

[31] Krause F, Boldt J. Care in healthcare: reflections on theory and practice. 2017. 31314223

[32] Mol A. The logic of care: Health and the problem of patient choice. 2008: Routledge. ISBN: 0203927079.

[33] Fisher B, et al. Toward a feminist theory of caring. Family: Crit Concepts Sociol. 1990;2:29–54.

[34] Held V. The ethics of care: Personal, political, and global. Oxford university press.ISBN; 2005. p. 0198040008.

[35] Tronto JC. Who cares? How to reshape a democratic politics. Cornell University Press.ISBN; 2015. p. 1501702769.

[36] Tronto JC. An ethic of care. Generations: J Am Soc Aging. 1998;22(3):15–20. 12785337

[37] Verbeek P-P. Acting artifacts: The technological mediation of action. User Behavior and Technology Development: Shaping Sustainable Relations Between Consumers and Technol. Springer; 2006. pp. 53–60. 10.1007/978-1-4020-5196-8_6.

[38] Hofmann B. Moral obligations towards human persons’ wellbeing versus their suffering: an analysis of perspectives of moral philosophy. Health Policy. 2024;142:105031. 38428058 10.1016/j.healthpol.2024.105031

[39] Wang S, Gupta M. Deontological ethics by monotonicity shape constraints. in International conference on artificial intelligence and statistics. 2020. PMLR.

[40] Beauchamp T, Childress J. Principles of biomedical ethics: marking its fortieth anniversary. Taylor & Francis; 2019. pp. 9–12. 10.1080/15265161.2019.1665402 31647760

[41] Dale S. A critique of principlism: virtue and the adjudication problem in bioethics. Voices Bioeth. 2023;9. 10.52214/vib.v9i.10522.

[42] Liu T, et al. A foundational triage system for improving accuracy in moderate acuity level emergency classifications. Commun Med. 2025;5(1):322. 40745473 10.1038/s43856-025-01052-w PMC12314101

[43] Heuser S, Steil J, Salloch S. AI ethics beyond principles: strengthening the life-world perspective. Sci Eng Ethics. 2025;31(1):7. 39928281 10.1007/s11948-025-00530-7 PMC11811459

[44] D’Alessandro W. Deontology and safe artificial intelligence. Philos Stud. 2024. 10.1007/s11098-024-02174-y.

[45] Hursthouse R. On virtue ethics, in Applied ethics. Routledge; 2017. pp. 29–35.

[46] Doukas DJ, et al. Virtue and care ethics & humanism in medical education: a scoping review. BMC Med Educ. 2022;22(1):131. 10.1186/s12909-021-03051-6. 35219311 10.1186/s12909-021-03051-6 PMC8881825

[47] Kristjánsson K, et al. Phronesis (practical wisdom) as a type of contextual integrative thinking. Rev Gen Psychol. 2021;25(3):239–57.

[48] Vallor S. Technology and the virtues: A philosophical guide to a future worth wanting. Oxford University Press.ISBN; 2016. p. 019049851X.

[49] Rochi M, et al. Technology paternalism: Development and validation of a measurement scale. Psychol Mark. 2024;41(5):1172–88. 10.1002/mar.21971.

[50] Vearrier L, Henderson CM. Utilitarian principlism as a framework for crisis healthcare ethics. in HEC forum. Springer; 2021. 10.1007/s10730-020-09431-7 PMC7809094 33449232

[51] Fu B, Hadid A, Damer N. Generative AI in the context of assistive technologies: Trends, limitations and future directions. Image Vis Comput. 2025;154. 10.1016/j.imavis.2024.105347.

[52] Savulescu J, Wilkinson D. Consequentialism and the Law in Medicine. Philosophical foundations of medical law; 2019. 31790169

[53] Obermeyer Z, et al. Dissecting racial bias in an algorithm used to manage the health of populations. Science. 2019;366(6464):447–53. 31649194 10.1126/science.aax2342

[54] Khanna NN, et al. Economics of artificial intelligence in healthcare: diagnosis vs. treatment. in Healthcare. MDPI; 2022. 10.3390/healthcare10122493 PMC9777836 36554017

[55] Zafar M. Normativity and AI moral agency. AI Ethics. 2025;5(3):2605–22.

[56] Ayers JW, et al. Comparing physician and artificial intelligence chatbot responses to patient questions posted to a public social media forum. JAMA Intern Med. 2023;183(6):589–96. 37115527 10.1001/jamainternmed.2023.1838 PMC10148230

[57] Rubin M, et al. Considering the Role of Human Empathy in AI-Driven Therapy. JMIR Ment Health. 2024;11:e56529. 10.2196/56529. 38861302 10.2196/56529 PMC11200042

[58] Kurian N. AI’s empathy gap: The risks of conversational Artificial Intelligence for young children’s well-being and key ethical considerations for early childhood education and care. Contemp Issues Early Child. 2023;26(1):132–9. 10.1177/14639491231206004.

[59] Kittay EF. The ethics of care, dependence, and disability. Ratio juris. 2011;24(1):49–58.

[60] Coeckelbergh M. Moral appearances: emotions, robots, and human morality. Ethics Inf Technol. 2010;12(3):235–41.

[61] Floridi L. AI and Semantic Pareidolia: When We See Intelligent Consciousness where there is None. Philos Technol. 2026;39(1). 10.1007/s13347-026-01052-1.

[62] Bergamin E, Roeser S. Technological mediation of emotional practices: The case of AI in healthcare. J Human-Technology Relations. 2025;3:1–18.

[63] Søraa RA, et al. What do older adults want from social robots? a qualitative research approach to human-robot interaction (HRI) studies. Int J Social Robot. 2023;15(3):411–24.

[64] Ragno L, et al. Application of social robots in healthcare: Review on characteristics, requirements, technical solutions. Sensors. 2023;23(15):6820. 37571603 10.3390/s23156820 PMC10422563

[65] Persson M, Iversen C, Redmalm D. Making robots matter in dementia care: Conceptualising the triadic interaction between caregiver, resident and robot animal. Volume 46. Sociology of Health & Illness; 2024. pp. 1192–211. 6. 10.1111/1467-9566.13786 38733615

[66] Rashid NLA, et al. The effectiveness of a therapeutic robot,‘Paro’, on behavioural and psychological symptoms, medication use, total sleep time and sociability in older adults with dementia: a systematic review and meta-analysis. Int J Nurs Stud. 2023;145:104530. 37348392 10.1016/j.ijnurstu.2023.104530

[67] Guemghar I, et al. Social robot interventions in mental health care and their outcomes, barriers, and facilitators: scoping review. JMIR Mental Health. 2022;9(4):e36094. 35438639 10.2196/36094 PMC9066335

[68] Hung L, et al. Ethical considerations in the use of social robots for supporting mental health and wellbeing in older adults in long-term care. Front Rob AI. 2025;12:1560214. 10.3389/frobt.2025.1560214 PMC11994439 40231303

[69] Hurmuz MZ, et al. Are social robots the solution for shortages in rehabilitation care? Assessing the acceptance of nurses and patients of a social robot. Computers Hum Behavior: Artif Hum. 2023;1(2):100017.

[70] Leoste J, et al. Evaluating Social Assistive Robots in Clinical Nursing Care: Mixed Method Pilot Study on Health Care Workers’ Perceptions and Adoption. JMIR Nurs. 2025;8:e70305. 40749105 10.2196/70305 PMC12319256

[71] Lee OE, et al. My precious friend: Human-robot interactions in home care for socially isolated older adults. Clin Gerontologist. 2024;47(1):161–70. 10.1080/07317115.2022.2156829 36502295

[72] Shin H, Lee OE. Who is behind the robot? The role of public social workers in implementing robotic eldercare program in South Korea. Soc Work Health Care. 2024;63(4–5):311–27. 38448245 10.1080/00981389.2024.2324849

[73] Sparrow R, Sparrow L. In the hands of machines? The future of aged care. Mind Mach. 2006;16(2):141–61.

[74] Tronto J. Moral boundaries: A political argument for an ethic of care. 2020: Routledge. ISBN: 1003070671.

[75] Miles A, et al. Robotic Animal Use among Older Adults Enrolled in Palliative or Hospice Care: A Scoping Review and Framework for Future Research. Robotics. 2024;13(6):92.

[76] Ocagli H, et al. In-bed monitoring: A systematic review of the evaluation of in-bed movements through bed sensors. in Informatics. MDPI; 2024.

[77] Kulurkar P, et al. AI based elderly fall prediction system using wearable sensors: A smart home-care technology with IOT. Volume 25. Measurement: Sensors; 2023. p. 100614.

[78] Schiff GD. AI-Driven Clinical Documentation—Driving Out the Chitchat? New England Journal of Medicine; 2025. 10.1056/NEJMp2416064 40353584

[79] Sjoding MW, et al. Racial bias in pulse oximetry measurement. N Engl J Med. 2020;383(25):2477–8. 33326721 10.1056/NEJMc2029240 PMC7808260

[80] Villegas-Galaviz C, Martin K. Moral distance, AI, and the ethics of care. AI Soc. 2023;1–12. 10.1007/s00146-023-01642-z. 10.1007/s00146-023-01642-z PMC10033285 37358944

[81] González A, Iffland C. Care professions and globalization: Theoretical and practical Perspectives. 2014: Springer.ISBN: 1137376481.

[82] Yuan S, et al. Ethical design of social robots in aged care: A literature review using an ethics of care perspective. Int J Social Robot. 2023;15(9):1637–54.

[83] Turkle S. Reclaiming conversation: The power of talk in a digital age. Penguin.ISBN: 110161739X; 2015.

